# Genome-wide analysis of mRNAs, lncRNAs, and circRNAs during intramuscular adipogenesis in Chinese Guizhou Congjiang pigs

**DOI:** 10.1371/journal.pone.0261293

**Published:** 2022-01-25

**Authors:** Lulin Tan, Zhaojun Chen, MingDe Teng, Bin Chen, Houqiang Xu

**Affiliations:** 1 College of Life Science, Guizhou University, Guiyang, China; 2 Key Laboratory of Animal Genetics, Breeding and Reproduction in the Plateau Mountainous Region, Ministry of Education, Guizhou University, Guiyang, China; 3 Guizhou Animal Husbandry and Veterinary Research Institute, Guizhou Academy of Agricultural Sciences, Guiyang, China; 4 The Potato Institute of Guizhou Province, Guizhou Academy of Agricultural Sciences, Guiyang, China; University of Bologna, ITALY

## Abstract

Intramuscular fat content is an important determinant of meat quality, and preadipocyte differentiation plays a critical role in intramuscular fat deposition in pigs. However, many types of RNA differentiation, including messenger RNA (mRNA), long non-coding RNA (lncRNA), and circular RNA (circRNA) remain unreported despite their crucial roles in regulating adipogenesis. Chinese Guizhou Congjiang pigs are raised in the Guizhou province of China for their high-quality meat. Therefore, it is important for breeders to explore the mechanisms of proliferation and differentiation of intramuscular adipocytes from the *longissimus dorsi* muscle of these pigs. In the present study, a transcriptome analysis of intramuscular preadipocytes from Chinese Guizhou Congjiang pigs, including analyses of mRNAs, lncRNAs, and circRNAs at days 0 (D0), 4 (D4), and 8 (D8) was performed. A total of 1,538, 639, and 445 differentially expressed (DE) mRNAs, 479, 192, and 126 DE lncRNAs, and 360, 439, and 304 DE circRNAs were detected between D4 and D0, D8 and D0, and D8 and D4, respectively. Functional analyses identified many significantly enriched RNAs related to lipid deposition, cell differentiation, metabolism processes, and obesity-related diseases, biological processes, and pathways. We identified two lncRNAs (TCONS_00012086 and TCONS_00007245) closely related to fat deposition according to their target genes and tissue expression profiles. Subcellular distribution analysis using quantitative real-time PCR (qRT-PCR) revealed that both TCONS_00012086 and TCONS_00007245 are cytoplasmic lncRNAs. These data provide a genome-wide resource for mRNAs, lncRNAs, and circRNAs potentially involved in Chinese Guizhou Congjiang pig fat metabolism, thus improving our understanding of their function in adipogenesis.

## Introduction

Intramuscular fat content is positively correlated with flavor, tenderness, and juiciness in pork and is closely related to pork quality [1,2]. Therefore, understanding the mechanism of intramuscular fat formation would facilitate the improvement of meat quality. Differentiation and lipid accumulation differ between intramuscular and subcutaneous preadipocytes due to their differing glucose utilization and lipid metabolism mechanisms [3]. Porcine adipocyte growth is closely related to adipose tissue expansion [4], therefore understanding and controlling intramuscular preadipocyte differentiation may help to regulate intramuscular fat.

In recent years scientists have identified thousands of non-coding RNAs in humans, plants, fish, insects, and mammals due to advances in sequencing technologies and bioinformatic approaches [5]. Two new categories of non-coding RNAs, long non-coding RNAs (lncRNAs) and circular RNAs (circRNAs), were found to play vital roles in various biological processes [6,7]. LncRNAs, non-coding transcripts longer than 200 nucleotides, are key regulators of gene expression at the transcriptional and post-transcriptional levels [1,2]. Muñoz *et al*. [8] identified six lncRNAs in Iberian pigs with expression levels positively correlated to intramuscular fat content. Wang *et al*. [7] analyzed the expression of lncRNAs in subcutaneous adipose tissue from castrated and intact full-sib pairs of Huainan male pigs and found 18 differentially expressed lncRNAs that may play a role in fat deposition and have target genes involved in fatty acid, insulin, and the adipocytokine signaling pathway. These studies indicate that lncRNAs may determine fat deposition and fatty acid composition, and/or regulate adipogenic differentiation and lipid metabolism [9]. CircRNAs were discovered in pathogens during the 1970s [6] and have a variety of functions in physiological processes including lipid metabolism, obesity, hypertension, and cardiovascular disease [10]. Several studies have focused on circRNA expression profiles in humans [11], mammals [12], and chickens [13]. However, there is limited research on the expression profiles and functions of lncRNAs and circRNAs in intramuscular preadipocytes from Chinese indigenous pig breeds during different stages of differentiation.

Chinese Guizhou Congjiang pigs are a heritage breed that is largely restricted to the Guizhou Province and is raised primarily for meat. Their meat is high-quality, fragrant, and tender due to its high intramuscular fat content. However, RNA expression profiles of these pigs during intramuscular preadipocyte differentiation have not yet been reported. Therefore, in the present study, mRNA, lncRNA, and circRNA expression were detected using RNA sequencing (RNA-Seq) during different stages of intramuscular preadipocyte differentiation (days 0 (D0), 4 (D4), and 8 (D8)). This research can help us to understand the involvement of RNAs in intramuscular fat.

## Materials and methods

### Animals

Three 3-day-old Chinese Guizhou Congjiang piglets were obtained from a local livestock farm. All animal care and experimental procedures were approved by the Guizhou University Animal Care and Use Committee, Guizhou, China. The ethics approval document number was EAE-GZU-2021-P004.

### Intramuscular preadipocyte culture and differentiation

Three piglets were killed via venous administration of sodium pentobarbital (30 mg/kg of body weight) and sterilized with 75% ethanol to obtain samples of the *longissimus dorsi* muscle. The tissue was then washed with phosphate-buffered saline (PBS) three times and cut into 1–2 cm pieces. The pieces were digested with 2 mg/mL collagenase type Ⅰ at 37°C for 65 min and shaken well every 10 min. The digested tissue was diluted with an equal volume of DMEM/F12 growth medium and filtered with gauze to remove any undigested tissues. The filtered solution was then strained using 200 and 400 μm cell strainers and centrifuged at 1500 r/min for 10 min to collect the progenitor cells. The cells were cultured in DMEM/F12 growth medium containing 10% fetal bovine serum (FBS) and 1% penicillin-streptomycin at 37°C in an atmosphere of 5% CO_2_. Because preadipocytes attach much earlier than myoblasts, the cultured cells were washed with PBS three times in order to remove unadherent cells and insoluble myofibrillar proteins after culturing for two hours [14]. After the preadipocytes reached confluence (D0), the growth medium was substituted with induction medium (growth medium supplemented with 5 mM IBMX, 1 μM DEX, and 5 μg/mL insulin). After two days, the cells were cultured in maintenance medium (growth medium supplemented with 5μg/mL insulin) for an additional two days (D4), and the medium was changed every two days until day eight (D8). Three samples were collected on days 0, 4, and 8 for sequencing.

### Oil Red O staining and triglyceride analysis

The cultured cells were washed three times with PBS prior to Oil Red O staining, and then fixed in 4% paraformaldehyde for 30 min. The stained cells were again washed three times with PBS and observed under an inverted microscope (Nikon, Tokyo, Japan). Triglyceride levels were measured using a commercial assay kit (Nanjing Jiancheng Bioengineering Institute, Nanjing, China).

### RNA extaction, library preparation, and sequencing

Total RNA was isolated on D0, D4, and D8 using the Trizol reagent (Takara, Dalian, China). RNA integrity was assessed using an RNA Nano 6000 Assay Kit in a Bioanalyzer 2100 system (Agilent Technologies, CA, USA) and 1% agarose gel electrophoresis. The RNA integrity numbers (RINs) for all samples were > 9.8. RNA concentration was measured using a Qubit® RNA Assay Kit in a Qubit® 2.0 Fluorometer (Life Technologies, CA, USA).

Ribosomal RNA was removed using a Epicentre Ribo-Zero™ rRNA Removal Kit (Epicentre, USA), and sequencing libraries were generated using an rRNA-depleted RNA by NEBNext® Ultra™ Directional RNA Library Prep Kit for Illumina® (NEB, USA) following manufacturers’ recommendations. Briefly, fragmentation was carried out using divalent cations under elevated temperature in NEBNext First Strand Synthesis Reaction Buffer (5X). First strand cDNA was synthesized using random hexamer primer and M-MuLV Reverse Transcriptase (RNaseH-). Second strand cDNA synthesis was subsequently performed using DNA polymerase I and RNase H. dNTPs with dTTP were replaced by dUTP in the reaction buffer and any remaining overhangs were converted into blunt ends via exonuclease/polymerase activity. After adenylation of 3’ends of DNA fragments, NEBNext Adaptors with hairpin loop structure were ligated to prepare for hybridization. In order to select cDNA fragments 150–200 bp in length, the library fragments were purified using an AMPure XP system (Beckman Coulter, Beverly, USA). Next, 3μl USER Enzyme (NEB, USA) was added to size-selected, adaptor-ligated cDNA and processed at 37°C for 15 min and at 95°Cfor 5 min. PCR was then performed using Phusion High-Fidelity DNA polymerase, Universal PCR primers, and Index (X) Primer. Nine strand-specific libraries were sequenced on an Illumina Hiseq 4000 platform at Novogene Corporation (Beijing, China). The flowchart of library construction is shown in S1 Fig.

### Quality control and read mapping

Clean data (clean reads) used in downstream analyses were obtained by removing reads containing adapter, reads containing ploy-N, and low quality reads from the raw data. At the same time, the Q20, Q30, and GC content of the clean data were calculated. All of the downstream analyses were based on the clean data with high quality. The index of the reference genome was built and paired-end clean reads were aligned to the pig reference genome (*Sus scrofa* 11.1) using HISAT2 [15].

### Identification of lncRNA

All of the transcripts were merged using Cuffmerge software. LncRNAs were then identified from the assembled transcripts in four steps: (1) removal of lowly-expressed transcripts with FPKM < 0.5; (2) removal of transcripts with < 200 bp and < 2 exons; (3) removal of transcripts with protein-coding capability using the coding-non-coding index (CNCI), protein folding domain database (Pfam), coding potential calculator 2 database (CPC2), and phylogenetic codon substitution frequency (PhyloCSF); (4) removal of transcripts mapped within the 1 kb flanking regions of an annotated gene using Cuffcompare. Novel lncRNAs were named according to HGNC rules [16]. The flowchart of information analysis is shown in S2 Fig.

### Identification of circRNA

After the clean reads were aligned to the porcine reference genome, the junctions of the unmapped reads were identified using Findcirc software [17]. In brief, an index of the reference genome was built using Bowtie (v2.0.622). The unmapped reads were retained, and 20-mers sequences from the 5′ and 3′ ends of these reads were used to again align the reference genome using Bowtie. The anchored sequences were subsequently analyzed using find_circ software2, and the complete reads were aligned with breakpoints flanked by GT/AG splice sites. Back-spliced reads with > 2 read counts were identified as circRNAs. This process is detailed in S3(A) Fig.

Candidate circRNAs were then identified using the CIRI (circRNA identifier) algorithm [18]. In brief, paired chiastic clip-ping, paired-end mapping, and GT-AG splicing signals were discovered by scanning the slicing alignments obtained through the process detailed above. Next, the alignment files were scanned again using a dynamic programming algorithm for detecting additional junction reads and eliminating false positive circRNA candidates. The final circRNAs were obtained by retaining sequences with ≥ 2 junction reads. This process is detailed in S3(B) Fig.

Due to the high false positive rate in circRNA identification, the circRNA identified by the Findcirc software and CIRI were combined, and their intersection was retained for use.

### Differentially expressed (DE) genes and pathway analysis

The expression of mRNA and lncRNA was evaluated using fragments per kilobase of transcript sequenced per million base pairs sequenced (FPKM), and circRNA expression was estimated using TPM (transcripts per million). Differential expression of mRNAs, lncRNAs, and circRNAs was analyzed using the *DESeq2* R package, which identified DE genes as *p*_*adj*_ ≤ 0.05 and |log_2_(FoldChange)| > 1. The function of DE lncRNAs was predicted using Gene Ontology (GO) analysis of their co-location and co-expression target mRNAs. These were screened based on their genomic positional relation 100 kilobase pairs (kb) upstream and 100 kb downstream for co-location mRNAs and based on the Pearson correlation coefficient of lncRNA-RNA pairs (≥ 0.95) for co-expression mRNAs. The functions of DE circRNAs were analyzed using GO analysis of their parental genes. GO terms were performed for differentially expressed genes using GOseq [19], and p < 0.05 was considered significant. Kyoto Encyclopedia of Genes and Genomes (KEGG) pathway enrichment was evaluated using KOBAS (2.0) [20].

### Validation of DE genes by quantitative real-time RT-PCR

The primer pairs used in quantitative real-time RT-PCR (qRT-PCR) are listed in S1 Table. The RNA samples used in the sequencing were the same as those used in the qRT-PCR. The RNA was reversed to cDNA with RevertAid First Strand cDNA Synthesis Kit (Thermo, Waltham, USA). Then, qRT-PCR was performed using the SYBR Green PCR kit (Takara, Dalian, China) according to the manufacturer’s instructions with GAPDH (glyceraldehyde-3-phosphate dehydrogenase) as a reference gene. The expression levels of the target genes were calculated using the 2^−△△Ct^ method and the data were expressed as values relative to the D0 group.

### Subcellular localization

Porcine intramuscular preadipocytes were used for subcellular localization of lncRNA. Porcine intramuscular preadipocytes were lysed in cold lysis buffer and placed on ice for 10 min. Then, cells were centrifuged (12,000 g for 3 min, 4°C) and the supernatant retained as the cytoplasmic fraction and immediately frozen (80°C) for subsequent analysis. The nuclear pellet was resuspended with nuclear extraction buffer and placed on ice for 30 min, and then centrifuged (16,000 g for 5 min, 4°C). The supernatant was removed and the remainder (nuclear fraction) was frozen (80°C) for subsequent analysis.

### Statistical analysis

Data were analyzed using one-way ANOVAs in SPSS 18.0 statistical software. Differences were considered significant at *p*<0.05.

### Data accessibility

Our sequencing data have been submitted to the Sequence Read Archive database with accession number PRJNA718643.

## Results

### Phenotypic changes during intramuscular preadipocyte differentiation

**Fig 1:**
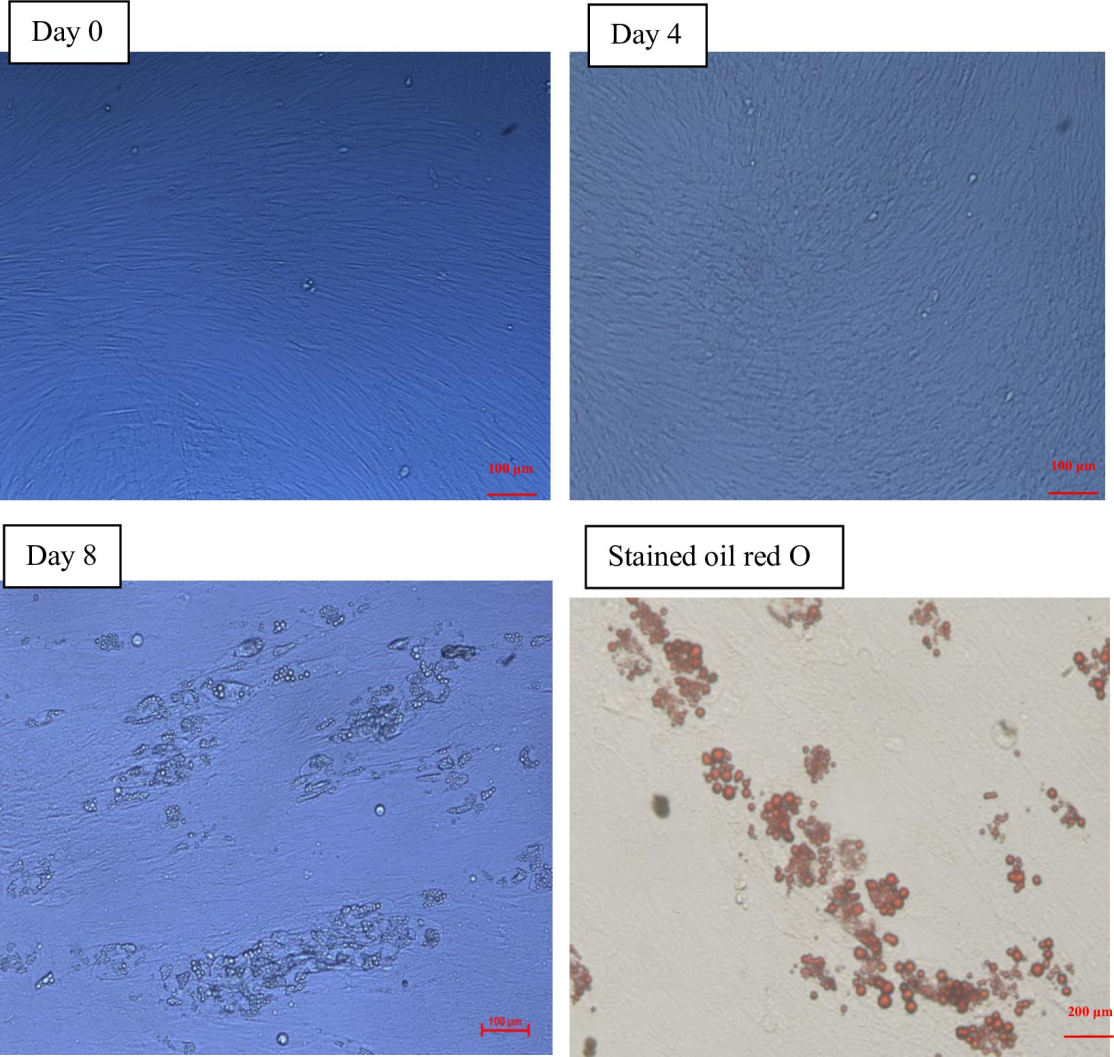
*In vitro* intramuscular preadipocyte differentiation. Intramuscular preadipocytes were obtained from the *longissimus doris* tissue of three three-day-old Chinese Guizhou Congjiang pigs and collected at three differentiation stages: Day 0, day 4, and day 8. Photos show enlarged adipocytes during differentiation (day 0, day 4, day 8; day 8 with Oil Red O staining).

**Fig 2:**
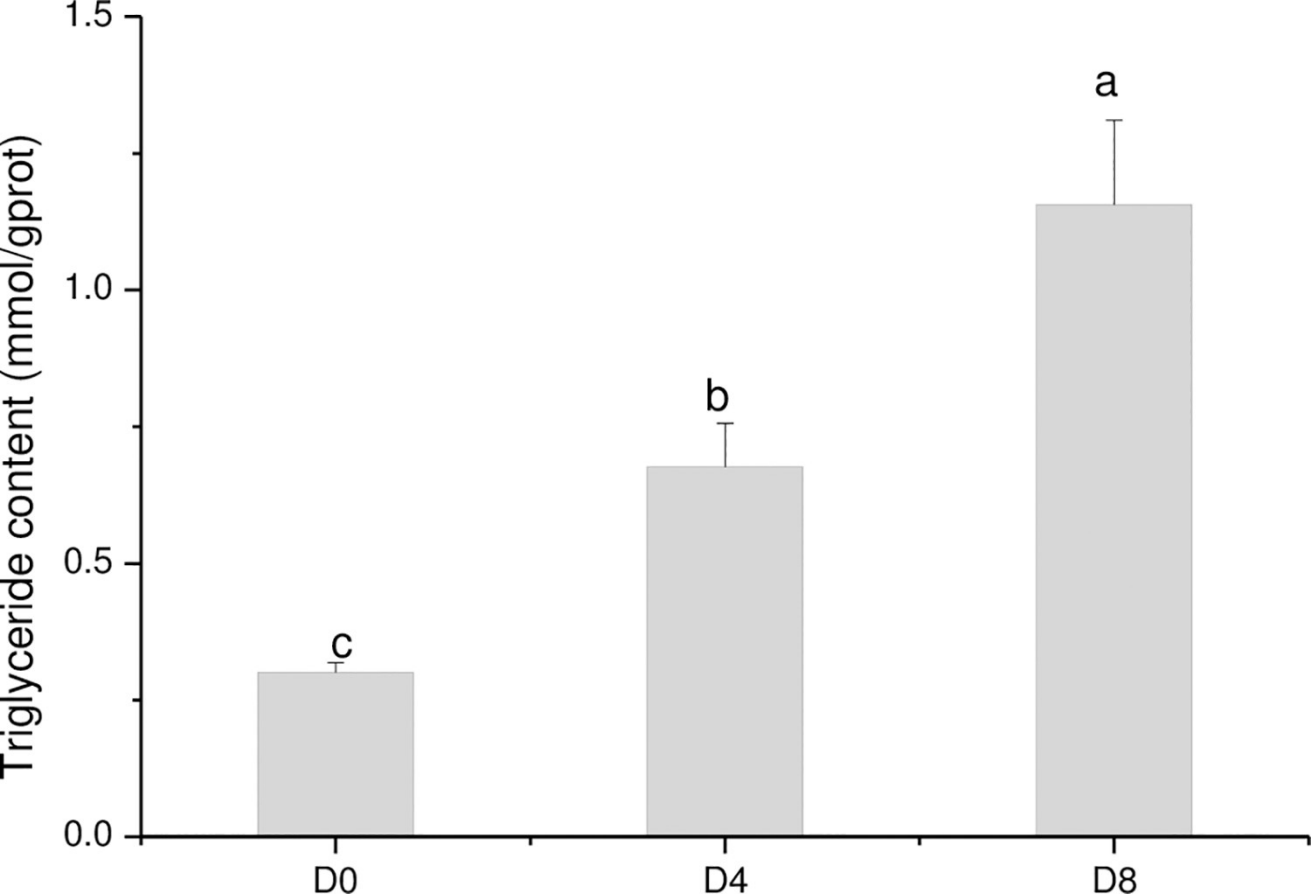
Triglyceride content at different stages of differentiation was measured using a triglycerol assay kit.

Preadipocytes changed from fibrous during the initial phase (D0) to spherical, and lipid droplets were visible on D4. The number of lipid droplets increased through D8 (Fig 1). The triglyceride content increased during preadipocyte differentiation (Fig 2). These data indicated that the intramuscular preadipocyte differentiation process was occurring as normal and could be further analyzed.

### Quality control and RNA sequencing

A total of 962,969,676 clean reads were obtained from all samples containing nine complementary libraries (S2 Table). The percentage of clean Q30 bases ranged from 90.59% to 92.06% (S2 Table). The clean reads were mapped to the pig reference genome with a ratio ranging from 90.18% to 93.21% (S2 Table). These results indicated that our data quality and mapping ratio were satisfactory for further analysis.

### Differentially expressed of mRNAs, lncRNAs, and circRNAs during intramuscular preadipocyte differentiation

**Fig 3:**
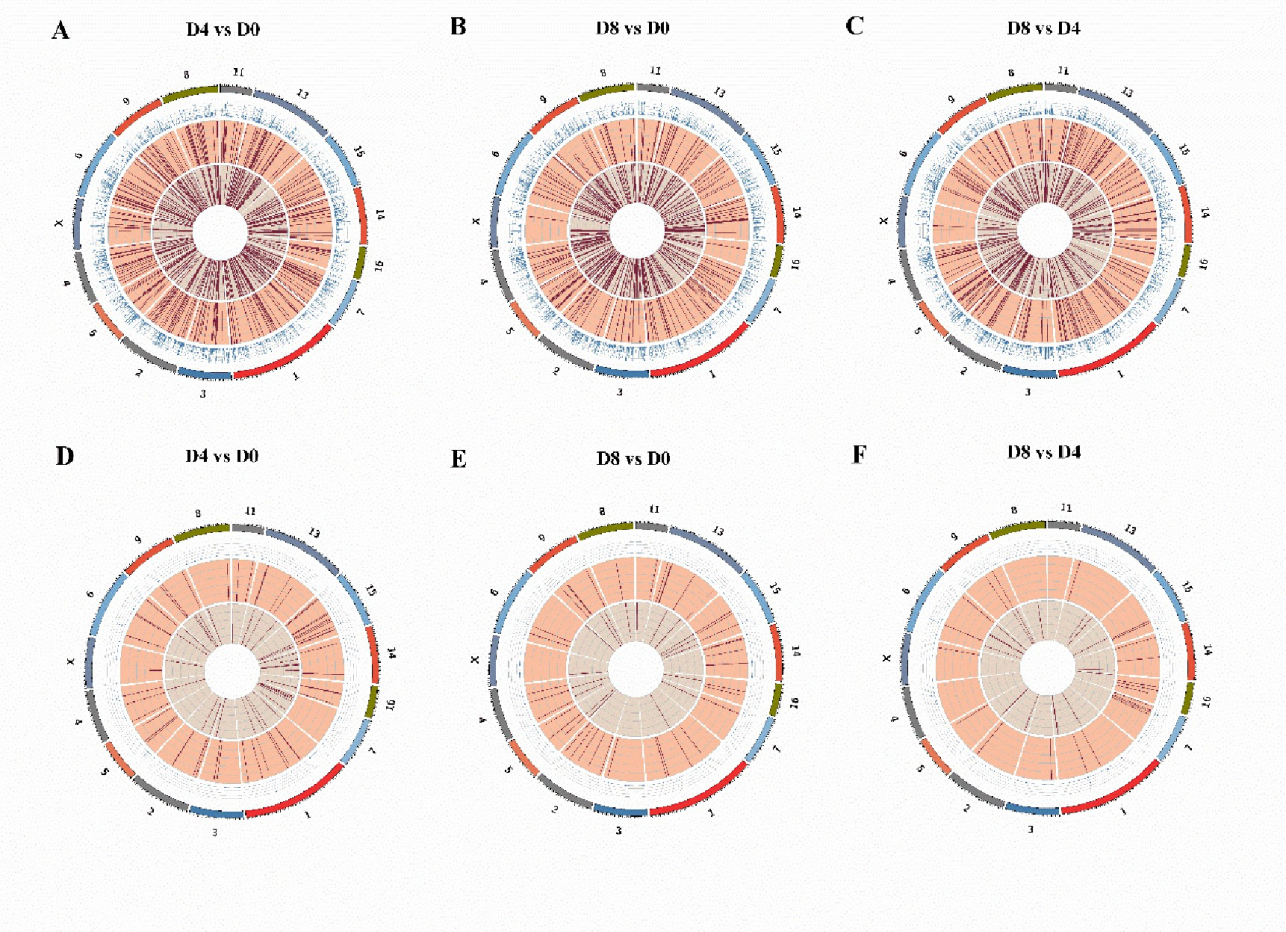
Circos map analysis of differentially distributed mRNA (A-C) and lncRNA (D-F).

**Fig 4:**
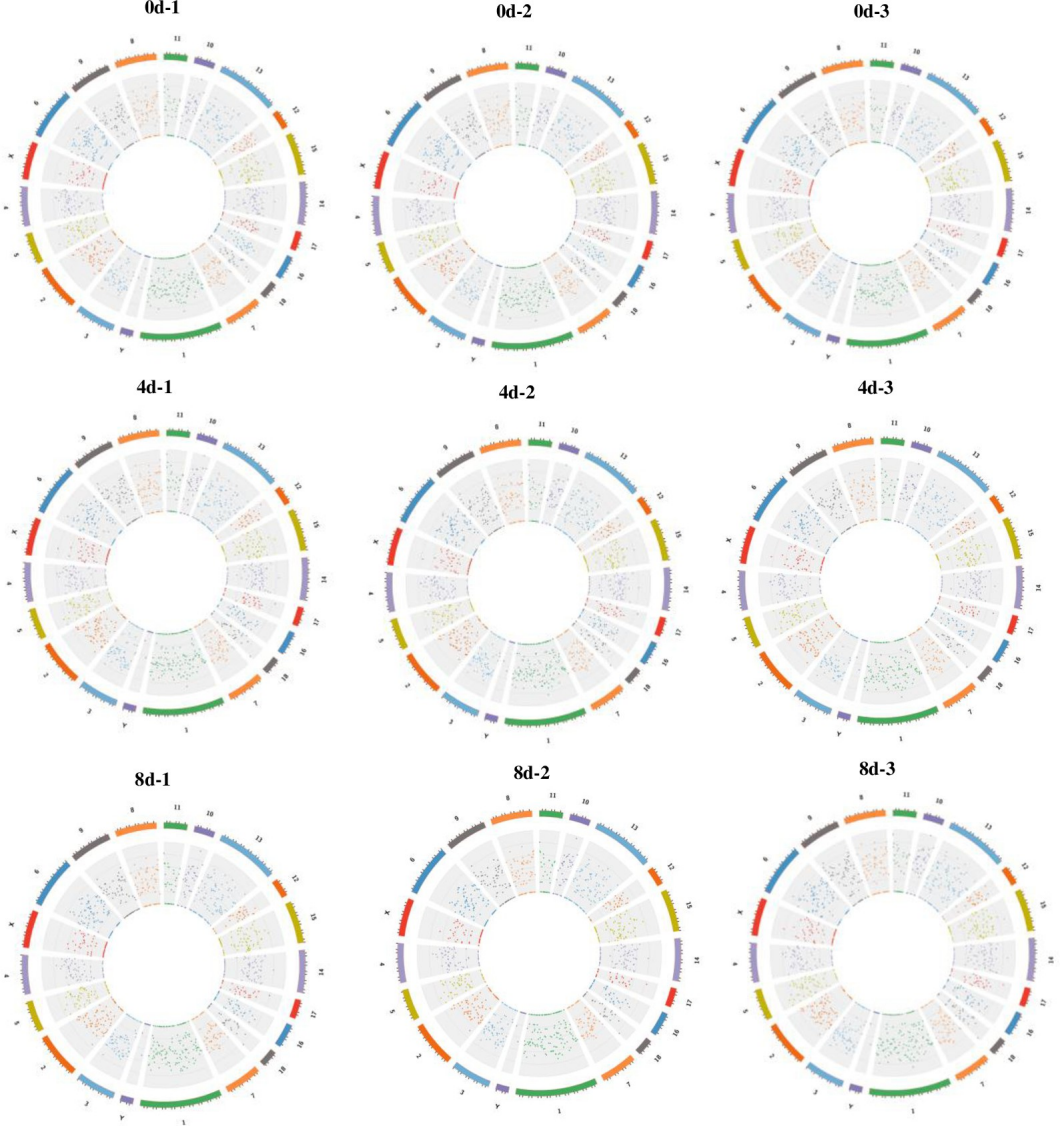
Circos map analysis of circRNA in each chromosome.

**Fig 5:**
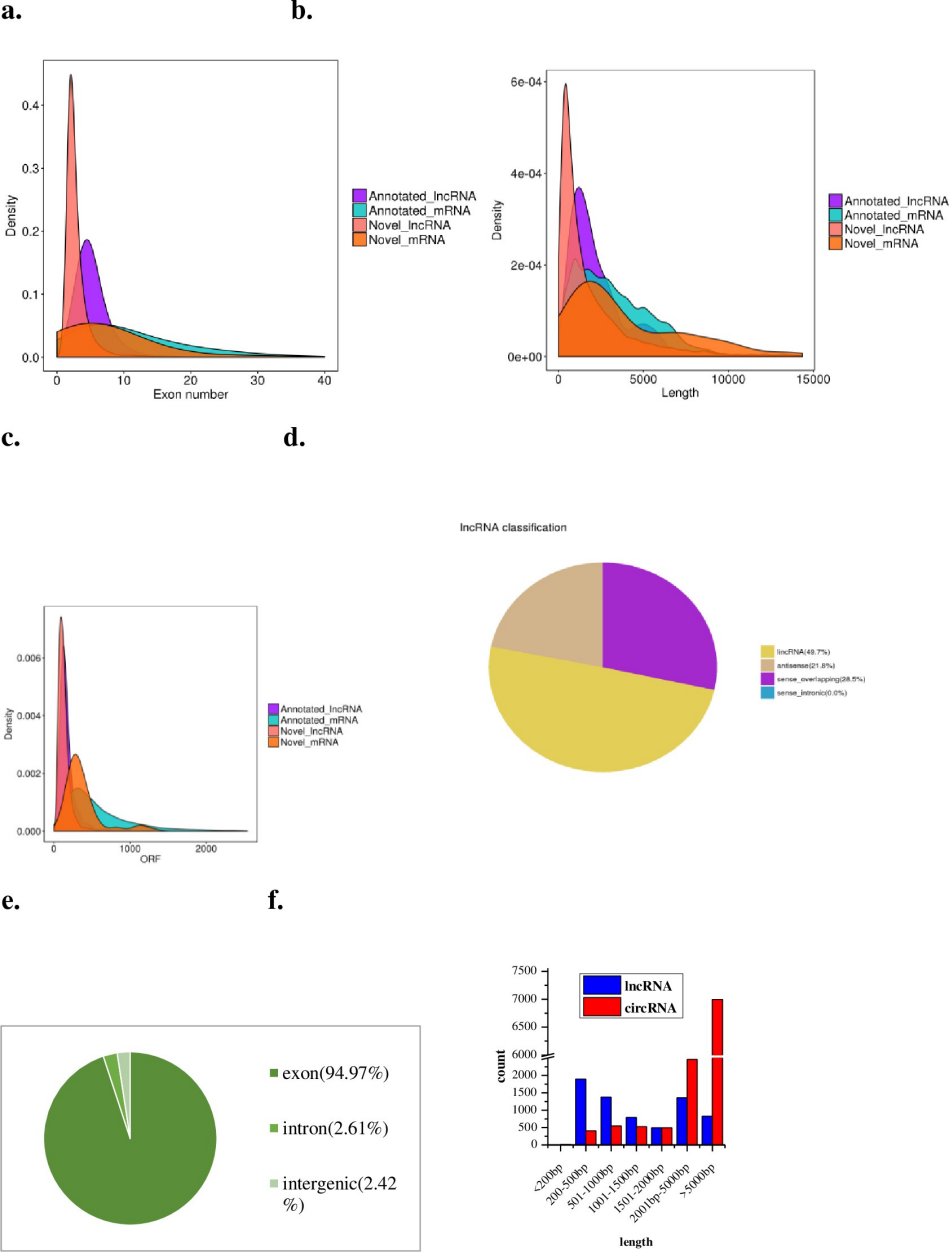
The features of lncRNAs and circRNAs during intramuscular preadipocyte differentiation. (a) Comparison of the numbers of exons in lncRNA and mRNA. (b) Comparison of the lengths of lncRNA and mRNA. (c) Comparison of the ORF sequence lengths of lncRNA and mRNA. (d) The types and proportions of novel lncRNAs. (e) The types and proportions of novel circRNAs. (f) The length distributions of lncRNAs and circRNAs.

The total numbers of mRNAs, lncRNAs, and circRNAs obtained from the three differentiation stages were 25,880, 4,278, and 11,439 respectively. A total of 9,940 mRNAs, 397 lncRNAs, and 1,284 cirRNAs (FPKM ≥ 1 in all samples) were detected as reliably expressed genes during intramuscular preadipocyte differentiation (S3 Table). Three comparisons (D4 vs D0, D8 vs D0, D8 vs D4) were used to quantify DE genes during intramuscular preadipocyte differentiation (Table 1). These comparisons revealed 1,538 mRNAs, 479 lncRNAs, and 360 circRNAs expressed differentially between D4 and D0 (Tables 1 and S4), 639 mRNAs, 192 lncRNAs, and 439 circRNAs expressed differentially between D8 and D0 (Tables 1 and S5), and 445 mRNAs, 126 lncRNAs, and 304 circRNAs expressed differentially between D8 and D4 (Tables 1 and S6). We found 33 mRNAs, 4 lncRNAs, and 11 circRNAs that were common differentially expressed genes throughout the differentiation process (S4(A)–S4(C) Fig). A circos map showing different distributions of mRNA and lncRNA in three comparisons is shown in Fig 3, and a circos map of circRNA distribution is shown in Fig 4. By comparing the transcript length, exon number, and ORF length, we showed that novel lncRNAs coincided with the general features of annotated lncRNAs (Fig 5A–5C). The lengths of the lncRNAs and circRNAs ranged from 201 to 116,890 bp and 158 to 95,950 bp, respectively. The majority (67.62%) of the lncRNAs was shorter than 2,000 bp, and approximately half of the identified circRNAs (61.1%) were longer than 5,000 bp (Fig 5F). The majority (49.7%) of novel lncRNAs was found in intergene region lncRNAs (“lincRNAs”), and the minority (21.8%) was antisense (Fig 5D). However, most (94.97%) novel circRNAs were exons, and only a small quantity were intergenic (2.42%) and intron circRNAs (2.61%) (Fig 5E).

**Table 1:** The number of DE mRNAs, lncRNAs, and circRNAs in the D4 vs. D0, D8 vs. D0, and D8 vs. D4 comparisons.

Groups	Upregulated mRNAs	Downregulated mRNAs	Upregulated lncRNAs	Downregulated lncRNAs	Upregulated circRNAs	Downregulated circRNAs
**D4 vs D0**	573	965	207	272	225	134
**D8 vs D0**	253	386	110	82	224	215
**D8 vs D4**	273	172	76	50	139	165

### Gene ontology and KEGG analysis of DE genes between D4 and D0

Between D4 and D0, we found significant GO terms for DE mRNA related to metabolic processes, primary metabolic processes, cellular component organization, intracellular processes, cytoplasmic parts, catalytic activity, and protein binding (S5(A) Fig). Based on DE lncRNAs with their trans-acting mRNAs, regulation of multicellular organisms, regulation of transport, immune system processes, membrane parts, intrinsic and integral membranes, receptor binding, long-chain fatty acid binding, fatty acid binding, carboxylic acid binding, and monocarboxylic acid binding were significantly enriched through GO analysis between D4 and D0 (S5(B) Fig). Based on DE lncRNAs with their cis-acting mRNAs, nucleus and intracellular parts were significantly enriched through GO analysis between D4 and D0 (S5(C) Fig). Significantly enriched GO terms were primarily involved in biological regulation, cellular and metabolic processes, growth, cell parts, binding, catalytic activity, enzyme regulator activity, and nucleic acid and protein binding transcription factor activity for DE circRNAs between D4 and D0 (S5(D) Fig).

**Fig 6:**
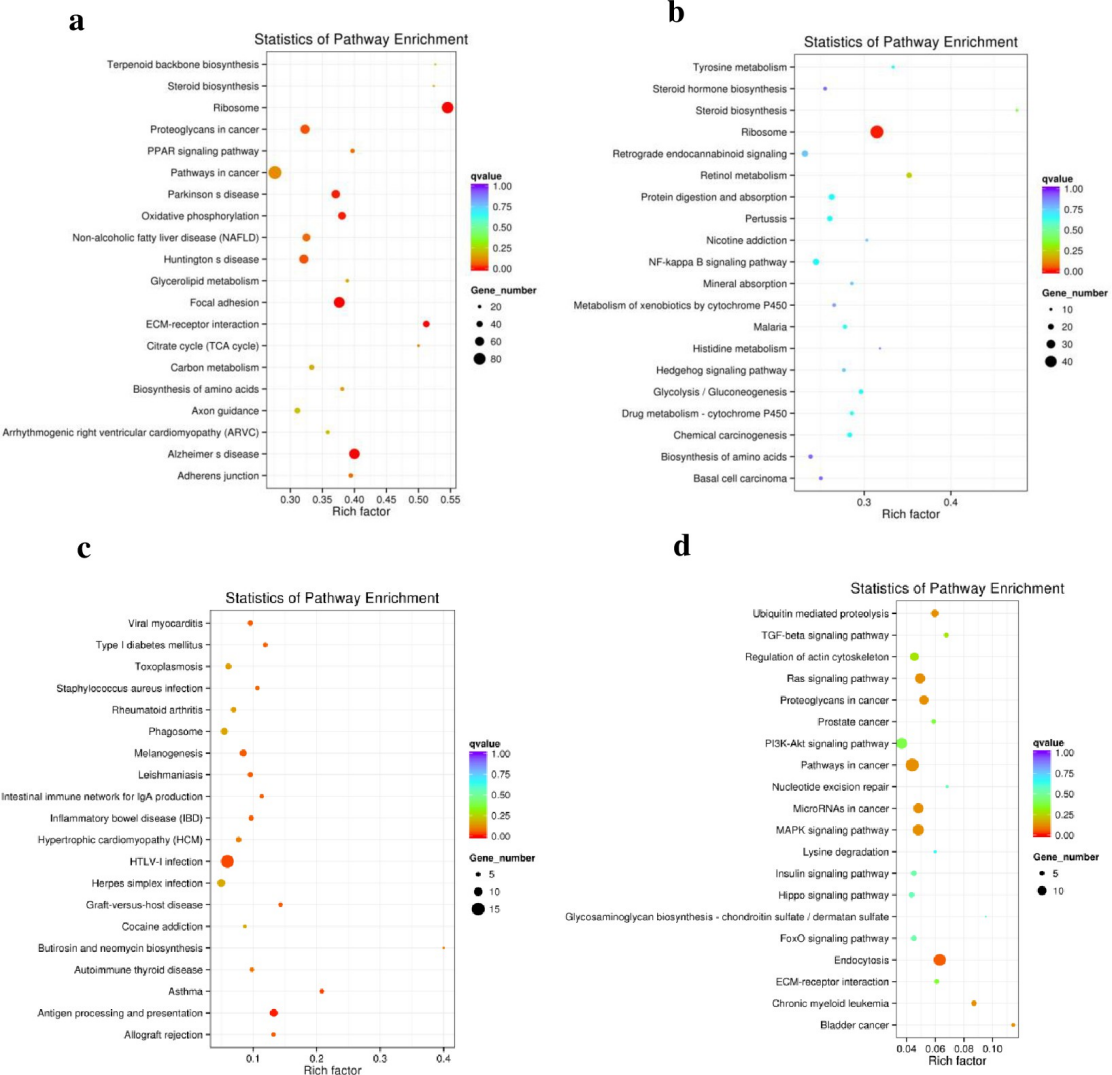
Kyoto Encyclopedia of Genes and Genomes (KEGG) enrichment analysis of differentially expressed genes between day 4 and day 0. (a) KEGG enrichment analysis of differentially expressed mRNAs. (b) KEGG enrichment analysis of differentially expressed lncRNAs with their trans-acting mRNA. (c) KEGG enrichment analysis of differentially expressed lncRNAs with their cis-acting mRNA. (d) KEGG enrichment analysis of differentially expressed circRNAs.

Between D4 and D0, significantly enriched KEGG terms for DE mRNAs were primarily involved in ribosomes, proteoglycans in cancer, PPAR signaling pathways, Parkinson’s disease, oxidative phosphorylation, non-alcoholic fatty liver disease, Huntington’s disease, focal adhesion, ECM-receptor interaction, Alzheimer’s disease, glycerolipid metabolism, and citrate cycles (Fig 6A). Significantly enriched KEGG terms for DE lncRNAs with their trans-acting mRNAs were primarily involved in ribosome and retinol metabolism (Fig 6B). Significantly enriched KEGG terms for lncRNAs with their cis-acting mRNAs were primarily involved in viral myocarditis, type Ⅰ diabetes mellitus, staphylococcus aureus infection, melanogenesis, leishmaniasis, the intestinal immune network for IgA production, inflammatory bowel disease (IBD), HTLV-I infection, and antigen processing and presentation (Fig 6C). Significantly enriched KEGG terms for DE circRNAs were primarily involved in ubiquitin-mediated proteolysis, ras signaling pathway, proteoglycans in cancer, pathways in cancer, the MAPK signaling pathway, and endocytosis (Fig 6D).

### Gene ontology and KEGG analysis of DE genes between D8 and D0

For DE mRNAs between D8 and D0, significant GO terms were found related to lipid metabolic processes, lipid biosynthetic processes, cardiovascular system development, growth factor binding, receptor binding, insulin-like growth factor binding, and protein binding (S6(A) Fig). Between D8 and D0, significant GO terms for DE lncRNAs with their trans-acting mRNAs were primarily involved in regulation of multicellular organisms, cell proliferation, cholesterol metabolic processes, regulation of immune system processes, collagen, membrane parts, receptor binding, cytoskeletal protein binding, and protein binding (S6(B) Fig). Significant GO terms for DE lncRNAs with their cis-acting mRNAs were primarily involved in the NF-kappa B complex, intracellular parts, nucleoplasm, extracellular space, the MHC protein complex, cytokine activity, receptor binding, and protein binding (S6(C) Fig). Significant GO terms for DE circRNAs were primarily involved in biological regulation, developmental processes, growth, metabolic processes, cell junctions, extracellular matrices, catalytic activity, enzyme regulator activity, and structural molecule activity (S6(D) Fig).

**Fig 7:**
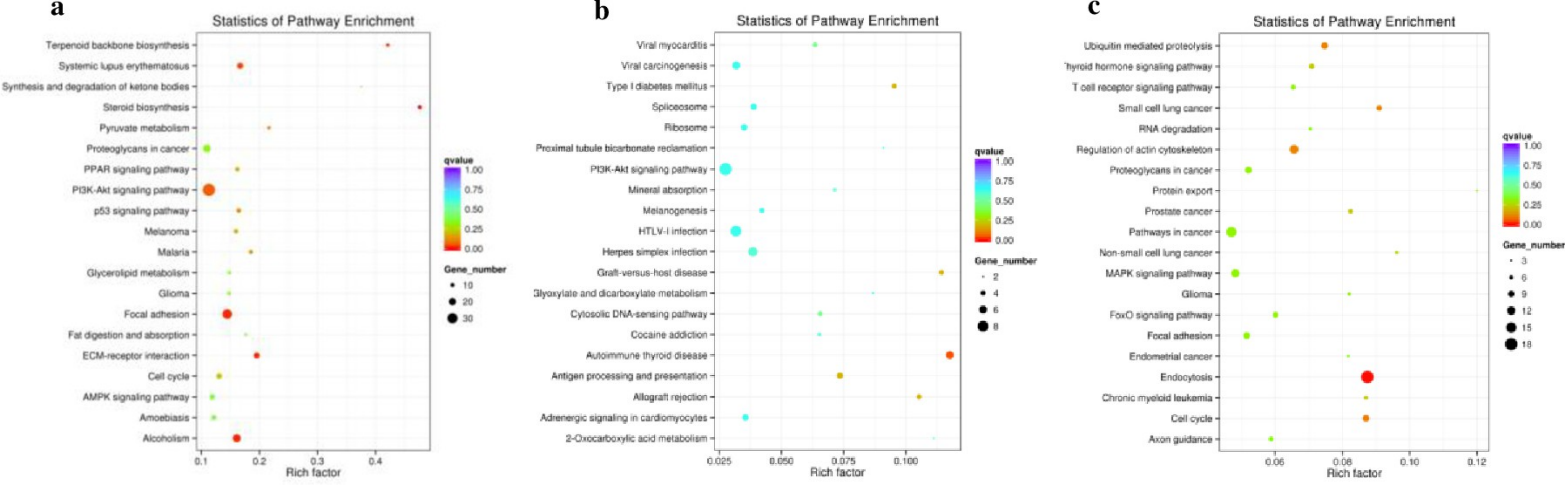
Kyoto Encyclopedia of Genes and Genomes (KEGG) enrichment analysis of differentially expressed genes between day 8 and day 0. (a) KEGG enrichment analysis of differentially expressed mRNAs. (b) KEGG enrichment analysis of differentially expressed lncRNAs with their cis-acting mRNA. (c) KEGG enrichment analysis of differentially expressed circRNAs.

Between D8 and D0, significantly enriched KEGG terms for DE mRNAs were primarily involved in terpenoid backbone biosynthesis, systemic lupus erythematosus, steroid biosynthesis, PI3K-Akt signaling pathways, focal adhesion, ECM-receptor interaction, and alcoholism (Fig 7A). Significantly enriched KEGG terms for DE lncRNAs with their cis-acting mRNAs were primarily involved in type Ⅰ diabetes mellitus, graft-versus-host disease, autoimmune thyroid disease, antigen procession and presentation, and allograft rejection (Fig 7B). Significantly enriched KEGG terms for DE circRNAs were primarily involved in ubiquitin-mediated proteolysis, small cell lung cancer, regulation of the actin cytoskeleton, endocytosis, and cell cycles (Fig 7C).

### Gene ontology and KEGG analysis of DE genes between D8 and D4

For DE mRNAs between D8 and D4, significant GO terms were found related to biological processes, cell adhesion, tissue development, regulation of responses to stimulus, regulation of signaling, and cytosolic parts (S7(A) Fig). Significant GO terms for DE lncRNAs with their trans-acting mRNAs were related to regulation of system processes, extracellular regions, plasma membrane parts, membrane-bounded vesicles, fatty-acid derivative binding, fatty-acid binding, long-chain fatty-acid binding, and enzyme inhibitor activity (S7(B) Fig). Significant GO terms for DE lncRNAs with their cis-acting mRNAs were related to regulation of cholesterol storage, sequence-specific DNA binding, muscle alpha-actin in binding, and interleukin-3 receptor activity (S7(C) Fig). Significant GO terms for DE circRNAs were related to biological regulation, cellular component organization or biogenesis, developmental processes, metabolic processes, responses to stimulus, cell junctions, catalytic activity, enzyme regulator activity, and translation regulator activity (S7(D) Fig).

**Fig 8:**
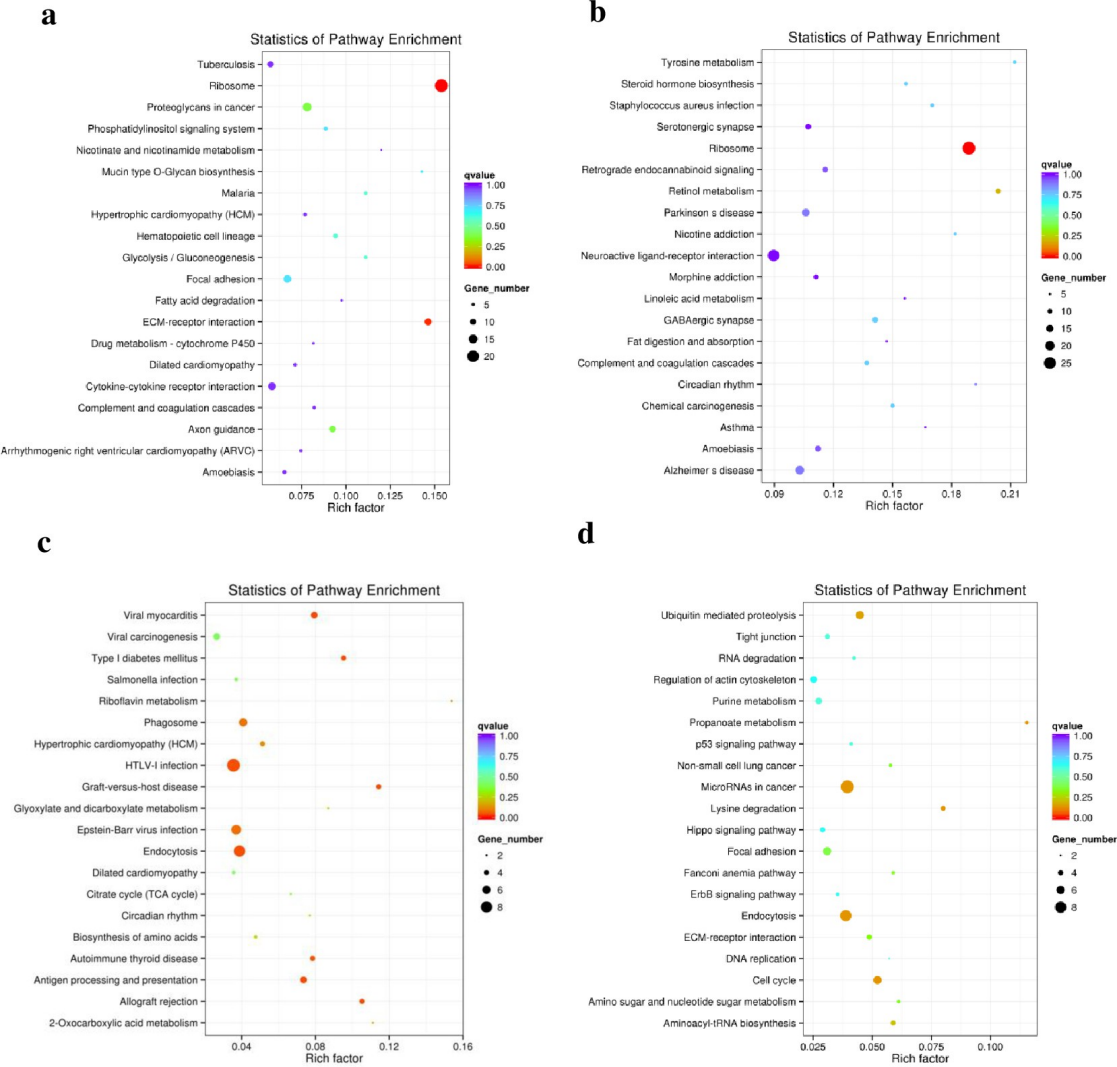
Kyoto Encyclopedia of Genes and Genomes (KEGG) enrichment analysis of differentially expressed genes between day 8 and day 4. (a) KEGG enrichment analysis of differentially expressed mRNAs. (b) KEGG enrichment analysis of differentially expressed lncRNAs with their trans-acting mRNA. (c) KEGG enrichment analysis of differentially expressed lncRNAs with their cis-acting mRNA. (d) KEGG enrichment analysis of differentially expressed circRNAs.

Between D8 and D4, significantly enriched KEGG terms for DE mRNAs were primarily involved in ribosomes, ECM-receptor interactions, the phosphatidylinositol signaling system, and fatty acid degradation (Fig 8A). Significantly enriched KEGG terms for DE lncRNAs with their trans-acting mRNAs were primarily involved in ribosomes, steroid hormone biosynthesis, linoleic acid metabolism, and fat digestion and absorption (Fig 8B). Significantly enriched KEGG terms for DE lncRNAs with their cis-acting mRNAs were primarily involved in viral myocarditis, type Ⅰ diabetes mellitus, phagosomes, hypertrophic cardiomyopathy (HCM), HTLV-Ⅰ infection, endocytosis, autoimmune thyroid disease, antigen processing and presentation, and allograft rejection (Fig 8C). Significantly enriched KEGG terms for DE circRNAs were primarily involved in ubiquitin-mediated proteolysis, regulation of actin cytoskeleton, propanoate metabolism, microRNAs in cancer, lysine degradation, endocytosis, and cell cycles (Fig 8D).

### Function analysis of common DE genes during intramuscular preadipocyte differentiation

Through comparison to the initial phase of differentiation (D0), 33 mRNAs, 4 lncRNAs, and 11 circRNAs were identified as common DE genes during the intramuscular preadipocyte differentiation process. Significant GO terms for DE mRNAs related to lipid metabolism and cell proliferation/differentiation were commonly significantly enriched based on comparisons between D4 and D0, D8 and D0, and D8 and D4 (Table 2). Significantly enriched KEGG terms for DE mRNAs were commonly involved in proteoglycans in cancer, focal adhesion, and ECM-receptor interaction based on comparisons between D4 and D0, D8 and D0, and D8 and D4. Significantly enriched KEGG terms for DE lncRNAs with their trans-acting mRNA were commonly involved in tyrosine metabolism, retrograde endocannabinoid signaling, retinol metabolism, and nicotine addiction during the intramuscular preadipocyte differentiation process. Significantly enriched KEGG terms for DE lncRNAs with their cis-acting mRNAs were commonly involved in viral myocarditis, Type Ⅰ diabetes mellitus, HTLV-Ⅰ infection, graft-versus-host disease, autoimmune thyroid disease, and allograft rejection based on comparisons between D4 and D0, D8 and D0, and D8 and D4. Significantly enriched KEGG terms for DE circRNA were commonly involved in ubiquitin-mediated proteolysis, regulation of actin cytoskeleton, and endocytosis based on comparisons between D4 and D0, D8 and D0, and D8 and D4.

**Table 2:** Summary of Gene Ontology (GO) analysis of differentially expressed mRNAs. Only significant terms are listed.

Groups	GO ID	Description	*p*-Value
**4d vs 0d**	GO:0048468	cell development	1.96E-06
	GO:0030154	cell differentiation	2.03E-06
	GO:0008283	cell proliferation	5.14E-06
	GO:0008610	lipid biosynthetic process	1.92E-05
	GO:0016049	cell growth	0.000305
	GO:0055088	lipid homeostasis	0.000511
	GO:0006629	lipid metabolic process	0.001068
**8d vs 0d**	GO:0008610	lipid biosynthetic process	1.23E-09
	GO:0006629	lipid metabolic process	4.98E-09
	GO:0007049	cell cycle	1.48E-05
	GO:0044255	cellular lipid metabolic process	1.51E-05
	GO:0045444	fat cell differentiation	6.63E-05
	GO:0006641	triglyceride metabolic process	7.46E-05
	GO:0019432	triglyceride biosynthetic process	0.00015818
	GO:0046463	acylglycerol biosynthetic process	0.00032991
	GO:0032787	monocarboxylic acid metabolic process	0.00051915
	GO:0022402	cell cycle process	0.00052275
	GO:0045598	regulation of fat cell differentiation	0.00085336
	GO:0006631	fatty acid metabolic process	0.00086921
	GO:0033993	response to lipid	0.00097827
**8d vs 4d**	GO:0000904	cell morphogenesis involved in differentiation	0.033362

### Validation of RNA-Seq by qRT-PCR

**Fig 9:**
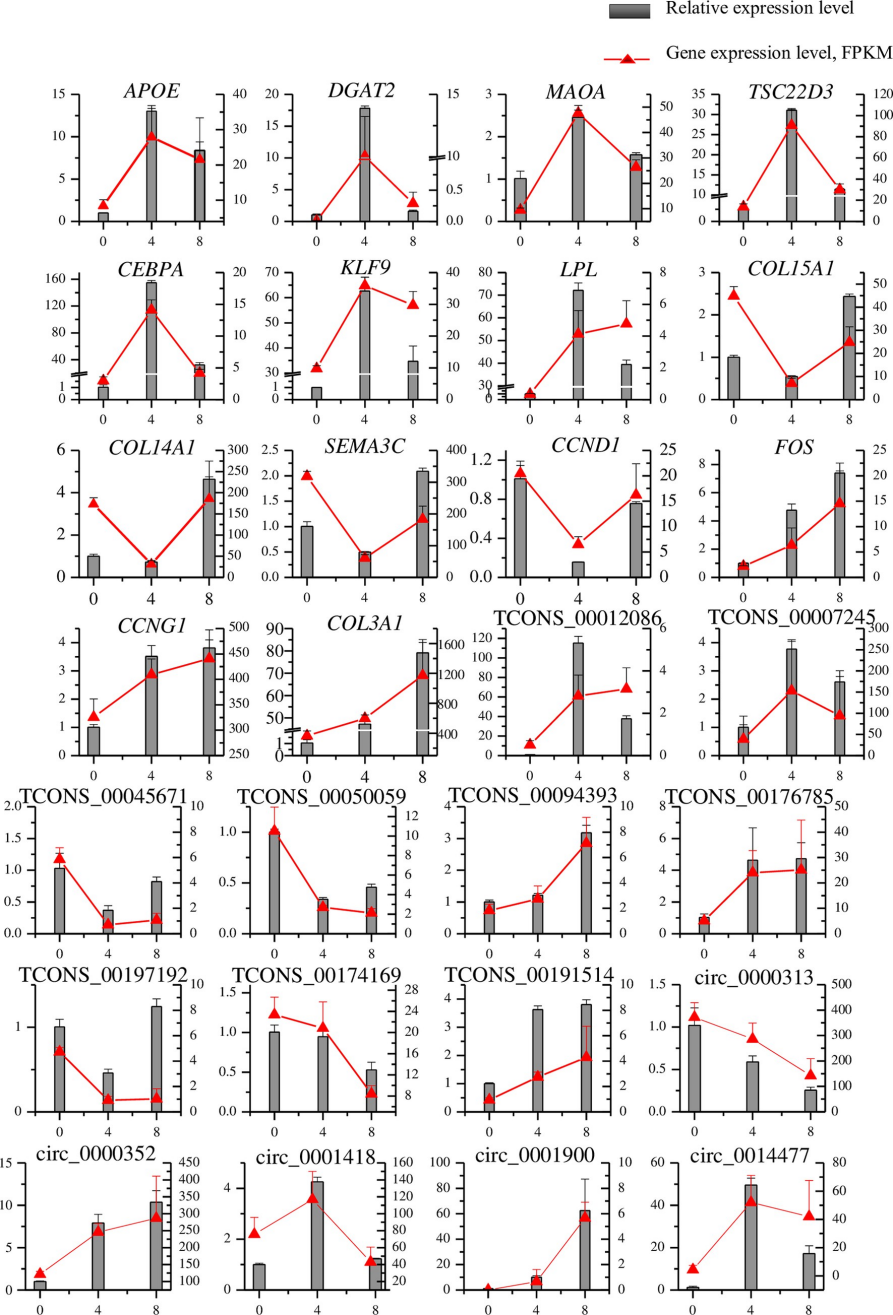
Validation of mRNAs, lncRNAs, and circRNAs involved in the three differentiation stages of lipid metabolism using reverse transcription qRT-PCR. Data from qRT-PCR are shown as columns and correspond to the Y-axis on the left, while the data from RNA-Seq are shown as a line and correspond to the Y-axis on the right. The error bars for qRT-PCR data and RNA-seq data represent standard errors.

QRT-PCR was used to validate the RNA-Seq data. We randomly selected 23 genes, including 14 mRNAs (*APOE*, *DGAT2*, *MAOA*, *TSC22D3*, *CEBPA*, *KLF9*, *LPL*, *COL15A1*, *COL14A1*, *SEMA3C*, *CCND1*, *FOS*, *CCNG1*, and *COL3A1*), 9 lncRNAs (TCONS_00012086, TCONS_00007245, TCONS_00045671, TCONS_00050059, TCONS_00094393, TCONS_00176785, TCONS_00197192, TCONS_00174169, and TCONS_00191514), and 5 circRNAs (circ_0000313, circ_0000352, circ_0001418, circ_0001900, and circ_0014477) to validate. The nine cell samples used in RNA-Seq were also used for qRT-PCR validation. Relative gene expression levels were calculated based on the mean value from nine cell samples using the comparative Ct method. After comparison with the RNA-Seq data, similar expression trends for qRT-PCR were discovered (Fig 9).

### Expression levels of candidate lncRNAs in different tissues

**Fig 10:**
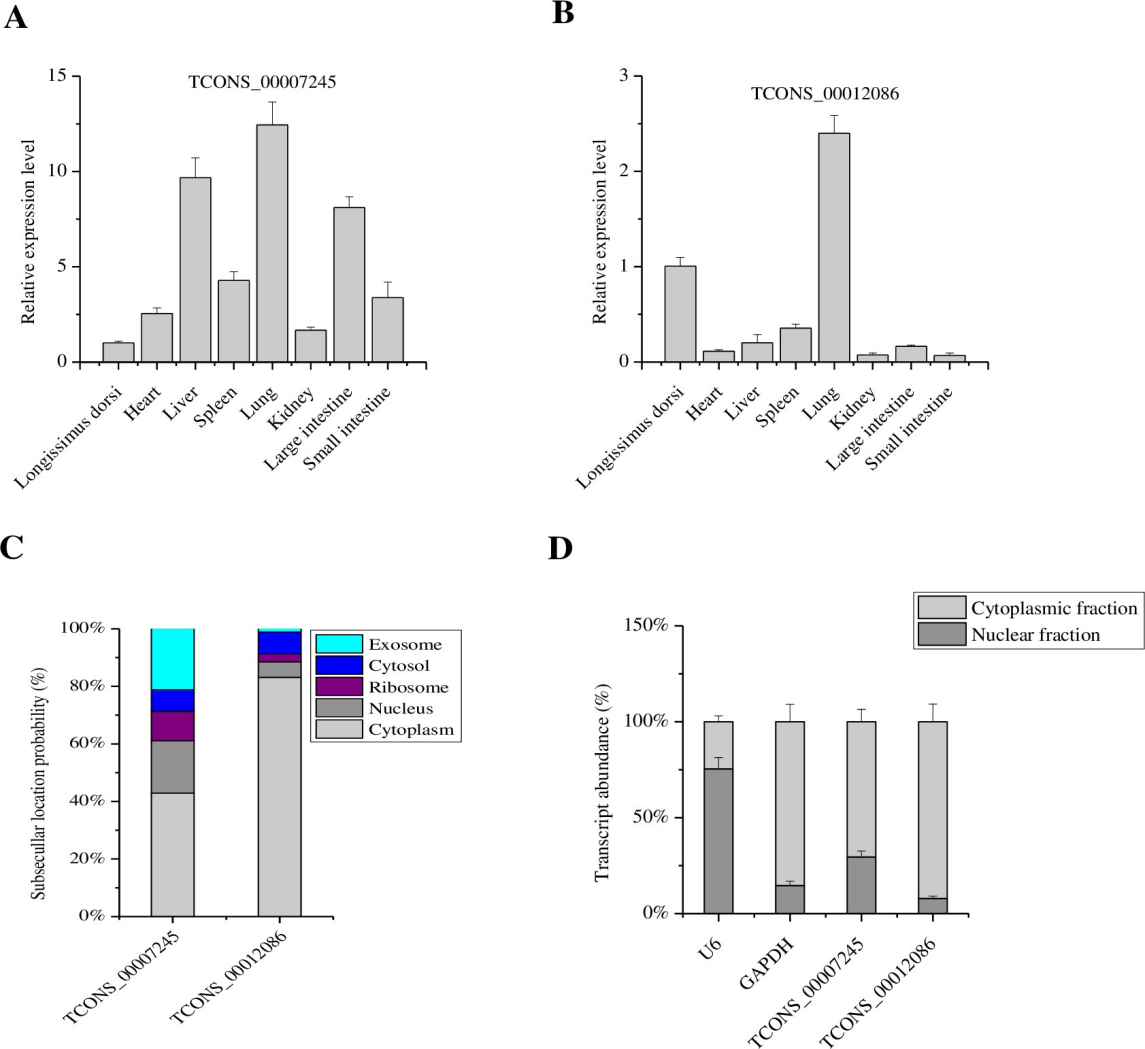
The relative expression levels of TCONS_00007245 (A) and TCONS_00012086 (B) in different Chinese Guizhou Congjiang pig tissues. (C) Prediction of subcellular localization by lncLocator (http://www.csbio.sjtu.edu.cn/bioinf/lncLocator/). (D) The nucleocytoplasmic fractionation of porcine cells by qRT-PCR. U6 RNA served as a nuclear location control and GAPDH served as a cytoplasmic location control.

QRT-PCR was used to explore the expression of TCONS_00012086 and TCONS_00007245 in Guizhou Congjiang pig tissues (Fig 10). TCONS_00012086 was most highly expressed in the lung tissue, followed by the *longissimus dorsi*, spleen, liver, large intestine, heart, small intestine, and kidney (Fig 10A). TCONS_00007245 was most highly expressed in lung tissues, followed by liver, large intestine, spleen, small intestine, heart, kidney, and *longissimus dorsi* (Fig 10B). Both prediction (Fig 10C) and qRT-PCR analysis (Fig 10D) suggested that TCONS_00012086 and TCONS_00007245 are located primarily in the cytoplasm of porcine intramuscular preadipocyte.

## Discussion

Pork quality is a key economic trait, and the molecular mechanisms underlying pork quality have important implications for breeding. We compared differences in the expression profiles of mRNAs, lncRNAs, and circRNAs during intramuscular preadipocyte differentiation in Chinese Guizhou Congjiang pigs for the first time. The shape of intramuscular adipocytes changed from shuttle-shaped to circular *in vitro* during the intramuscular preadipocyte differentiation process. Small lipid droplets were found on D4, and clusters of large lipid droplets were found on D8. Triglyceride content also increased gradually as intramuscular preadipocytes differentiated. Intramuscular preadipocyte differentiation is a complex process due to both adipocyte growth and lipid deposition. The general regulation of adipocyte growth and droplet formation observed in this study was consistent with previous research [21,22]. However, we observed that fat droplets were produced gradually beginning on D4, later than was observed in another study [22]. This difference may be related to the different pig breeds examined in the studies and/or to different types of preadipocytes. Furthermore, more than 465 DE mRNAs, 134 DE lncRNAs, and 139 DE circRNAs were detected during preadipocyte differentiation. Thus, our data provide a comprehensive understanding of the transcriptional regulation mechanisms driving the differentiation of intramuscular preadipocytes in Chinese Guizhou Congjiang pigs.

The intramuscular preadipocyte differentiation process consists of three critical stages, including growth arrest (D0), mitotic clonal expansion (D4), and late events and terminal differentiation (D8). DE mRNAs were enriched during lipid metabolism, cell proliferation, cell differentiation, cell development, and cell growth in the early stage of differentiation (D0 vs D4). At the same time, triglyceride content on D4 was significantly higher than on D0. The pathway of lipid metabolism and cell development/proliferation/growth was essential during the early stages of intramuscular preadipocyte differentiation, and this was consistent with other research [23]. According to the GO analyses, the pathways of lipid metabolic processes, lipid biosynthetic processes, fat cell differentiation, triglyceride metabolic processes, triglyceride biosynthetic processes, and fatty acid metabolic processes were significantly enriched on D8 relative to D0. Triglyceride content on D8 was significantly higher than on D0. The pathway of cell morphogenesis involved in differentiation was also significantly enriched on D8 relative to D4, and triglyceride content on D8 was significantly higher than on D4. These enrichment pathways are consistent with morphological changes and increased triglyceride levels during the intramuscular preadipocytes differentiation process. Therefore, these data support the claim that the changes in triglyceride and phenotype result from pathway enrichment of lipid lipolysis-related markers [24]. The expression of three adipose differentiation-related genes, *CEBPA*, *APOE*, and *LPL*, was significantly increased on D4. Transcription factor *CEBPA* is known to be a master gene that modulates the different stages of adipogenesis in adipocytes [25], and can couple induction of adipose differentiation-specific genes with cell cycle arrest, which is a necessary process for blocking cells during the G1 phase prior to preadipocyte differentiation [26]. The expression of *CCND1* decreased during the early stage of differentiation (D4 vs D0), consistent with results described in other research [27]. Our data demonstrated that *CEBPA* can impact growth arrest on D4 through downregulation of *CCND1* expression. *APOE* regulates lipid homeostasis by mediating lipid transport from one tissue or cell type to another, and positively correlates with triglyceride content [28]. *LPL* also plays a key role in lipid metabolism by catalyzing triglyceride hydrolysis of lipoproteins such as chylomicron and very-low-density lipoprotein (VLDL) into fatty acids [29]. The expression of fibril-associated collagens with interrupted triple helix-related genes *COL14A1* and *COL15A1* was significantly lower during the early stage of differentiation (D4 vs D0). *COL14A1* and *COL15A1* have an anti-proliferative role in reducing *de novo* DNA synthesis in 3T3-L1 preadipocytes [30]. Therefore, the decreased expression of *COL14A1* and *COL15A1* may be associated with the observed changes in intramuscular preadipocyte morphology.

Throughout the last decade, many studies have revealed the important roles that lncRNAs and circRNAs play in regulating adipocyte development, metabolic processes, and physiological processes [10,31]. Therefore, lncRNAs and circRNAs may represent a new approach to designing therapeutic and diagnostic methods [32,33]. A number of studies have reported differences in lncRNAs and circRNAs in the *longissimus dorsi* muscle of different porcine breeds [1,2,34]. Changes in lncRNA and circRNA expression during subcutaneous adipogenic differentiation in pigs have also been reported [21], whereas differences in lncRNAs and circRNAs in intramuscular adipogenesis in Chinese Guizhou Congjiang pigs have not been fully illustrated. The mechanisms through which lncRNAs and circRNAs influence intramuscular fat deposition also remain unclear. In the present study, we identified 397 lncRNAs and 1,284 circRNAs (FPKM or TPM ≥ 1 in all samples) as reliably expressed during intramuscular preadipocyte differentiation. We also detected 4 lncRNAs and 11 circRNAs that were shared differentially expressed genes. The characteristics of lncRNAs identified in present study are similar to those of rats, cows, humans, and other mammals, including fewer exons, shorter ORF sequence lengths and exon lengths, and lower expression levels than protein-coding genes [34,35]. These results can provide new insights into the mechanism of differentiation of intracellular adipocytes, and may also help us to understand the molecular mechanisms regulating lipid metabolism-related diseases in humans [36].

GO and KEGG pathway analyses were performed in order to explore differential expression of mRNAs during preadipocyte differentiation. As expected, many classical functional categories were found to be significantly enriched, including metabolic processes, intracellular parts, protein binding, biological processing, and regulation of responses to stimuli. Genes within these functional groups may play essential roles in the conversion of intramuscular preadipocytes to adipocytes [21].The PPAR signaling pathway was enriched on D4 relative to D0 and on D8 relative to D0, and is closely associated with lipid oxidation, fat cell differentiation, lipid droplet formation, and the activities of lipid metabolism-related enzymes during preadipocyte differentiation into adipocytes [37]. In addition, the PI3K-Akt signaling pathway was significantly enriched on D8 relative to D0. The PI3K-Akt signaling pathway plays a role in cell proliferation, differentiation, apoptosis, glucose metabolism, and lipid metabolism [38]. The p53 signaling pathway is related to initiating DNA repair, cell cycle arrest, senescence, and apoptosis [39]. The PPAR, PI3K-Akt, p53, and AMPK signaling pathways were significantly enriched for DE mRNAs on D8 relative to D0, similar to the results of previous studies [22].

The function of DE lncRNAs can be predicted from their cis- or trans-acting mRNAs, both of which may be important in the regulation of transcription and posttranscriptional modification. Trans target gene predictions were based on correlations between lncRNA and mRNA expression levels [31]. The main biochemical metabolic pathways and signal transduction pathways of the DE lncRNA-regulated target genes were identified using pathway enrichment. We found that retinol metabolism, ribosomes, steroid biosynthesis, the NF-kappa B signaling pathway, the FoxO signaling pathway, cell cycles, and fat digestion and absorption were significantly enriched. The NF-kappa B signaling pathway, as an important signaling cascade regulator, plays a role in innate immune response, alcoholic liver disease, DNA transcription regulation, and inflammatory gene expression [40]. The FoxO signaling pathway has a wide range of biological functions, including the regulation of cell proliferation, apoptosis, and differentiation [41,42]. That the NF-kappa B and FoxO signaling pathways were significantly enriched may provide a new insight for researching the function of lncRNA during intramuscular preadipocyte differentiation. LncRNA also regulates target genes via co-location [31], in which lncRNA in relatively close proximity to the protein-coding genes, and all genes in the proximity of the lncRNA loci (10 kb or 100 kb upstream or downstream) are target genes [34]. We carried out a functional enrichment analysis of differentially expressed lncRNA target genes during preadipocyte differentiation. We found many pathways involved in obesity and immune related diseases, including Type Ⅰ diabetes mellitus, hypertrophic cardiomyopathy (HCM), autoimmune thyroid disease, inflammatory bowel disease (IBD), and graft-versus-host disease. Although the function of lncRNAs in pigs has not been clearly shown, they are a valuable model animal in the study of obesity [34], and therefore this study provides a new view of lncRNAs that may help us to further understand their roles in translational and post-translational regulation of fat deposition and obesity-related diseases in humans.

GO and KEGG pathway analyses were performed to explore the function of DE circRNAs based on their genomic locations. As expected, many lipid metabolism-related functional categories were significantly enriched, including metabolic processes, enzyme regulator activities, and the regulation of biological processes. The MAPK signaling pathway was enriched on D4 relative to D0. The MAPK signaling pathway plays an important role in regulating adipocyte proliferation and differentiation in mammals because of the function of signal systems in mediating cell responses to external stimuli [6]. The cell cycle signaling pathway was significantly enriched on D8 vs D0 and D8 vs D4. This is not surprising because preadipocytes typically re-enter the cell cycle with at least one round of mitotic clonal expansion, increasing the proportion of adipocytes later in differentiation relative to the early stages [21]. The regulation of the actin cytoskeleton signaling pathway was significantly enriched on D8 relative to D0, likely because the actin cytoskeleton is closely related to lipid droplet formation and adipocyte maturation [33]. Interestingly, many signaling pathway-related diseases were enriched, including the ras signaling pathway, proteoglycans and microRNAs in cancer, and small cell lung cancer. These results may help us to better understand the relationships between circRNAs and those diseases [6].

In pig intramuscular preadipocytes, *XLOC_046142*, *XLOC_004398*, and *XLOC_015408* play main regulatory roles in adipogenesis and lipid accumulation by targeting *MAPKAPK2*, *NR1D2*, and *AKR1C4*, respectively [1]. LncRNA-PCAT1 promotes osteogenic differentiation by activating the TLR signaling pathway, which is negatively regulated by miR-145-5p in adipose-derived stem cells [9]. In the present study, two lncRNAs (TCONS_00012086 and TCONS_00007245) closely related to fat deposition according to their target genes were identified. Phosphodiesterase 7B (*PDE7B*) is the target gene of TCONS_00012086. Cyclic nucleotide phosphodiesterases (PDEs) are enzymes regulating cellular cAMP (or cGMP) concentration by controlling the rate of hydrolysis [43]. Increases in cellular cAMP concentration suppress Akt activity leading to cell growth arrest, apoptosis, and inhibition of adipose differentiation [43]. The novel gene *XLOC_005242*, first found in this study, is the target gene of TCONS_00007245, which was expressed at high levels in the liver. We speculate that TCONS_00007245 is related to lipid metabolism due to the important function of the liver in regulating lipid metabolism [44]. However, future research will be required to validate these predictions and explore the function of these lncRNAs.

## Conclusion

In conclusion, a genome-wide investigation of the expression of mRNAs, lncRNAs, and circRNAs during porcine intramuscular preadipocyte differentiation was undertaken. A large number of DE genes, which may contribute to phenotypic changes in adipocytes at different stages of differentiation, were identified. Our study provides a comprehensive record of the expression levels of various RNAs during adipocyte differentiation, and in turn provides a tool for better understanding the mechanisms underlying molecular regulation of intramuscular preadipocyte differentiation in pigs. Fourteen mRNAs (*DGAT2*, *MAOA*, *TSC22D3*, *CEBPA*, *KLF9*, *LPL*, *COL15A1*, *COL14A1*, *SEMA3C*, *CCND1*, *FOS*, *CCNG1*, and *COL3A1*), nine lncRNAs (TCONS_00012086, TCONS_00007245, TCONS_00045671, TCONS_00050059, TCONS_00094393, TCONS_00176785, TCONS_00197192, TCONS_00174169, and TCONS_00191514) and five circRNAs (circ_0000313, circ_0000352, circ_0001418, circ_0001900, and circ_0014477) were validated using qRT-PCR, and these were consistent with our RNA-seq results. Two lncRNAs (TCONS_00012086 and TCONS_00007245) that are closely related to fat deposition according to their target genes were identified, and their tissue expression profiles were detected. However, further study is necessary to identify their functions in pig preadipocytes. These findings provide a reliable foundation for future studies investigating the molecular mechanisms underlying preadipocyte differentiation in pigs.

## Supporting information

S1 Fig Flowchart of library construction. (DOC)

S2 Fig Flowchart of information analysis. (DOC)

S3 Fig Schematic diagram for circRNA discovery for find CIRC (a) and CIRI (b).
(DOC)

S4 Fig Venn diagram of DE mRNAs (a), lncRNAs (b), and circRNAs (c) at three time-points.
(DOC)

S5 Fig Gene Ontology (GO) functional enrichment analysis of differentially expressed genes between day 4 and day 0. (a) GO analysis of differentially expressed mRNAs. (b) GO analysis of differentially expressed lncRNAs with their trans-acting mRNA. (c) GO analysis of differentially expressed lncRNAs with their cis-acting mRNA. (d) GO analysis of differentially expressed circRNAs.
(DOC)

S6 Fig Gene Ontology (GO) functional enrichment analysis of differentially expressed genes between day 8 and day 0. (a) GO analysis of differentially expressed mRNAs. (b) GO analysis of differentially expressed lncRNAs with their trans-acting mRNA. (c) GO analysis of differentially expressed lncRNAs with their cis-acting mRNA between. (d) GO analysis of differentially expressed circRNAs.
(DOC)

S7 Fig Gene Ontology (GO) functional enrichment analysis of differentially expressed genes between day 8 and day 4. (a) GO analysis of differentially expressed mRNAs. (b) GO analysis of differentially expressed lncRNAs with their trans-acting mRNA. (c) GO analysis of differentially expressed lncRNAs with their cis-acting mRNA between. (d) GO analysis of differentially expressed circRNAs.
(DOC)

S1 Table Oligonucleotide primers used for quantitative real-time PCR of intramuscular preadipocytes. (XLSX)

S2 Table RNA-seq data from three stages of tramuscular preadipocyte differentiation. d0, d4 and d8 refer to three different time points during intramuscular preadipocyte differentiation (day 0, day 4, and day 8). 1, 2, and 3 refer to the three replicates.
(DOCX)

S3 Table The FPKM of all genes. (XLSX)

S4 Table The DE genes between D4 and D0. (XLSX)

S5 Table The DE genes between D8 and D0. (XLSX)

S6 Table The DE genes between D8 and D4. (XLSX)

## Abbreviations

mRNA: Messenger RNA
lncRNA: Long noncoding RNA
circRNA: Circular RNA
DE genes: Differentially expressed genes
