# Supplementary material for: Genome-wide analysis of mRNAs, lncRNAs, and circRNAs during intramuscular adipogenesis in Chinese Guizhou Congjiang pigs
Source: PLoS One. 2022 Jan 25;17(1):e0261293. doi: 10.1371/journal.pone.0261293 (PMC8789167; doi:10.1371/journal.pone.0261293)
Supplement: S2 Fig — (DOC) [file pone.0261293.s002.doc]

**S2 Figure.** Flowchart of information analysis.

**Known Transcript**

**Annotated lncRNA**

**Annotated mRNA**

**Novel Transcript**

**Novel mRNA**

**Novel lncRNA**

**unclassified**

**RNA sequencing**

**Quality control**

**Read mapping**

**Transcript splicing**

**Transcript screening**

**Quantitative analysis**

**Differential expression analysis**

**Differential lncRNA analysis**

**Differential mRNA analysis**

**Functional enrichment analysis**

**target genes**

**of lncRNA**

**GO**

**KEGG**
