# Supplementary material for: Genome-wide analysis of mRNAs, lncRNAs, and circRNAs during intramuscular adipogenesis in Chinese Guizhou Congjiang pigs
Source: PLoS One. 2022 Jan 25;17(1):e0261293. doi: 10.1371/journal.pone.0261293 (PMC8789167; doi:10.1371/journal.pone.0261293)
Supplement: S3 Fig — Schematic diagram for circRNA discovery for find CIRC (a) and CIRI (b). (DOC) [file pone.0261293.s003.doc]

**S3 Figure.** Schematic diagram for circRNA discovery for find CIRC (a) and CIRI (b).
