# Supplementary figures and images for: Genome-wide analysis of mRNAs, lncRNAs, and circRNAs during intramuscular adipogenesis in Chinese Guizhou Congjiang pigs
Source: PLoS One. 2022 Jan 25;17(1):e0261293. doi: 10.1371/journal.pone.0261293 (PMC8789167; doi:10.1371/journal.pone.0261293)

**S4 Figure.** Venn diagram of DE mRNAs (a), lncRNAs (b), and circRNAs (c) at three time-points.

**a. b.**

**
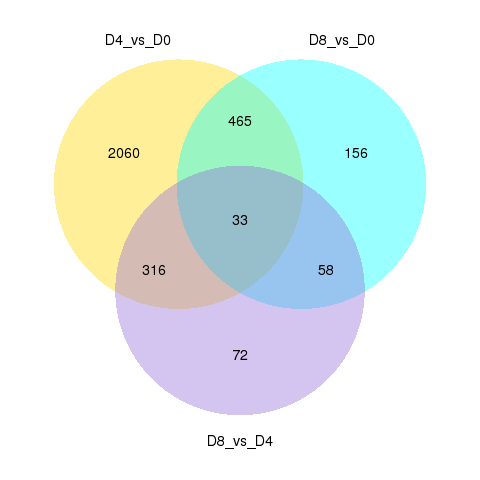

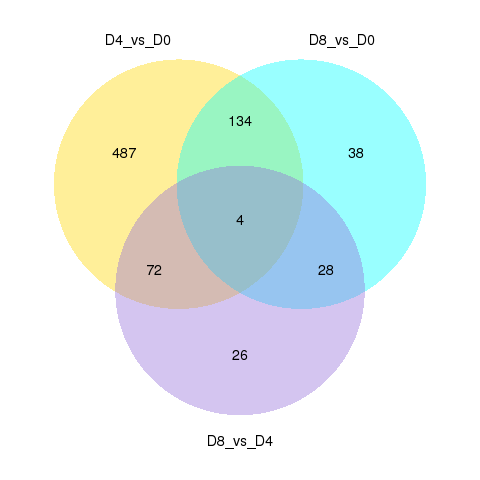
**

**c.**

**
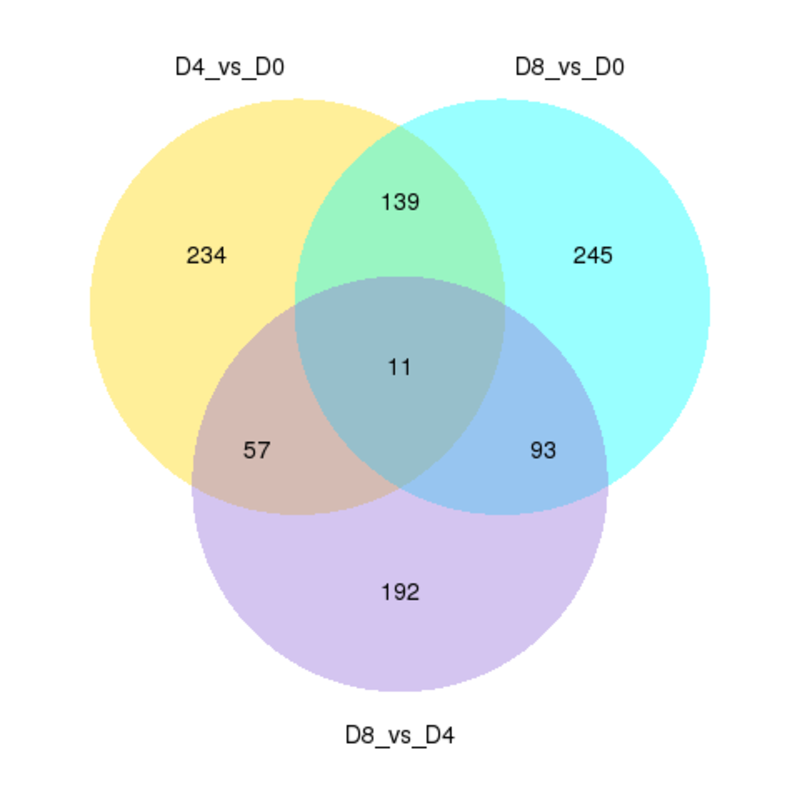
**

Supplement: S4 Fig — Venn diagram of DE mRNAs (a), lncRNAs (b), and circRNAs (c) at three time-points. (DOC) [file pone.0261293.s004.doc]
