# Supplementary material for: Genome-wide analysis of mRNAs, lncRNAs, and circRNAs during intramuscular adipogenesis in Chinese Guizhou Congjiang pigs
Source: PLoS One. 2022 Jan 25;17(1):e0261293. doi: 10.1371/journal.pone.0261293 (PMC8789167; doi:10.1371/journal.pone.0261293)
Supplement: S5 Fig — (a) GO analysis of differentially expressed mRNAs. (b) GO analysis of differentially expressed lncRNAs with their trans-acting mRNA. (c) GO analysis of differentially expressed lncRNAs with their cis-acting mRNA. (d) GO analysis of differentially expressed circRNAs. (DOC) [file pone.0261293.s005.doc]

**S5 Figure.** Gene Ontology (GO) functional enrichment analysis of differentially expressed genes between day 4 and day 0. (a) GO analysis of differentially expressed mRNAs. (b) GO analysis of differentially expressed lncRNAs with their trans-acting mRNA. (c) GO analysis of differentially expressed lncRNAs with their cis-acting mRNA. (d) GO analysis of differentially expressed circRNAs.

**
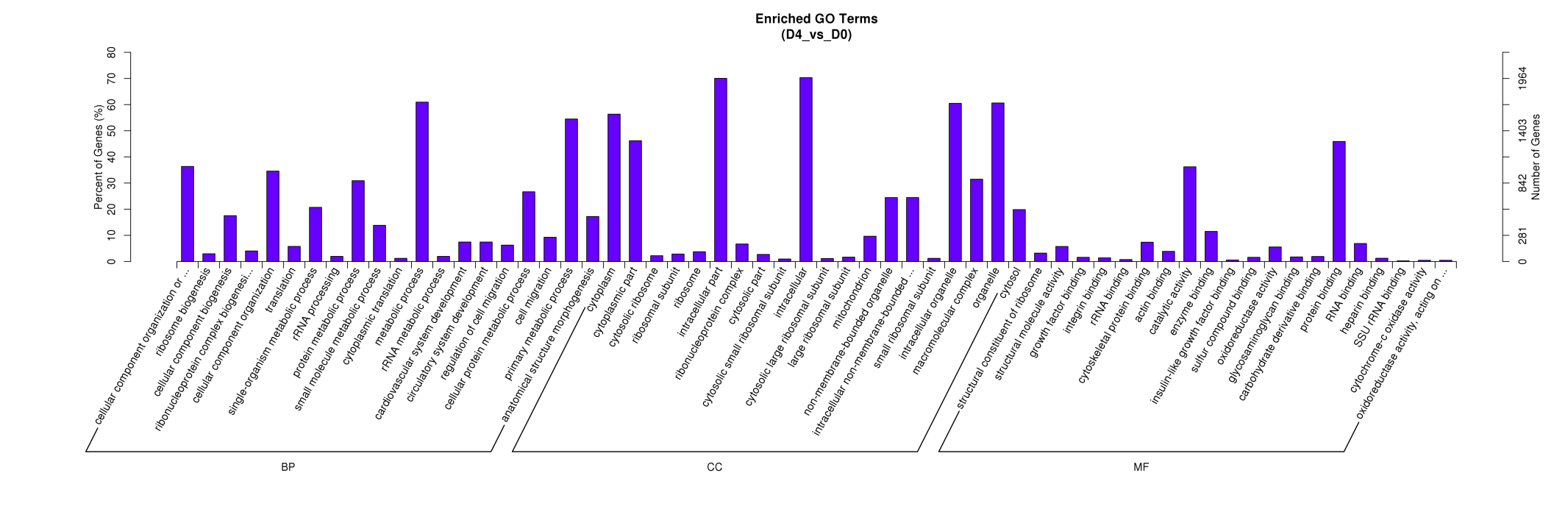
**

**b.**

**
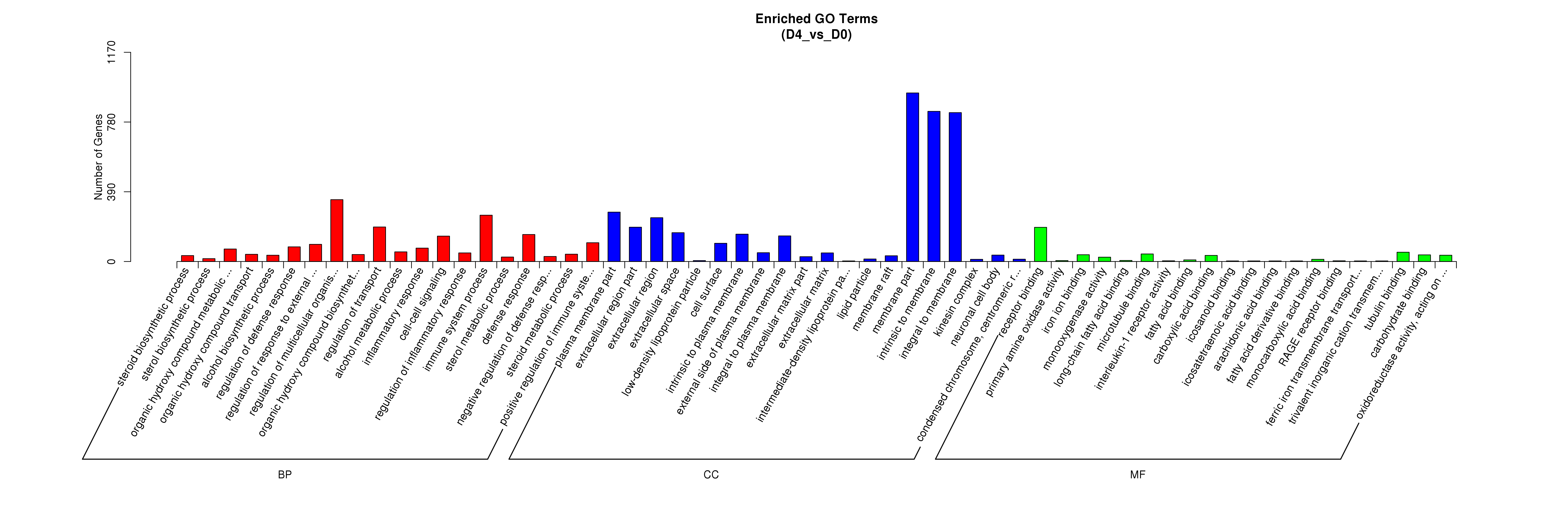
**

**c.**

**
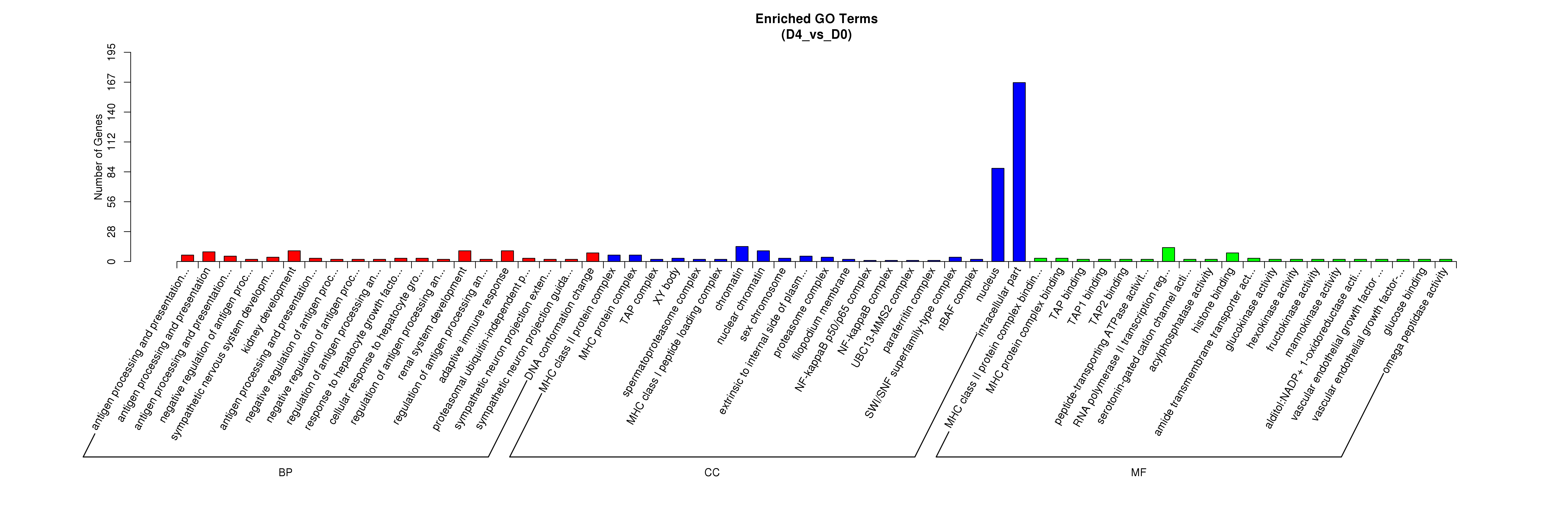
**

**d.**

**
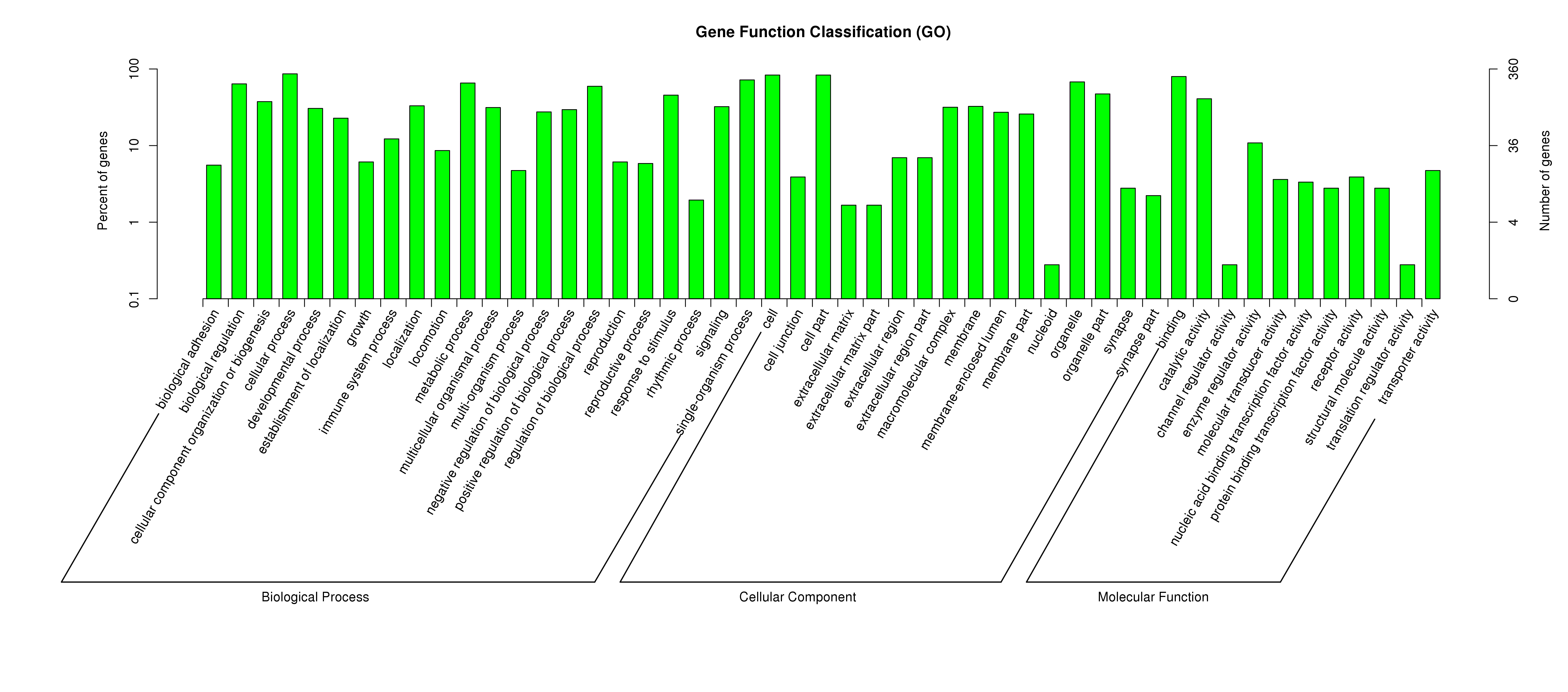
**
