# Supplementary material for: Genome-wide analysis of mRNAs, lncRNAs, and circRNAs during intramuscular adipogenesis in Chinese Guizhou Congjiang pigs
Source: PLoS One. 2022 Jan 25;17(1):e0261293. doi: 10.1371/journal.pone.0261293 (PMC8789167; doi:10.1371/journal.pone.0261293)
Supplement: S2 Table — d0, d4 and d8 refer to three different time points during intramuscular preadipocyte differentiation (day 0, day 4, and day 8). 1, 2, and 3 refer to the three replicates. (DOCX) [file pone.0261293.s009.docx]

**S2 Table.** RNA-seq data from three stages of tramuscular preadipocyte differentiation. d0, d4 and d8 refer to three different time points during intramuscular preadipocyte differentiation (day 0, day 4, and day 8). 1, 2, and 3 refer to the three replicates.

| **Sample name** | **Raw reads** | **Clean reads** | **Clean Q30 base rate (%)** | **Total mapped** | **Multiple mapped** | **Uniquely mapped** |
| --- | --- | --- | --- | --- | --- | --- |
| d0_1 | 130,216,660 | 129,213,378 | 90.95 | 119633343 (92.59%) | 6581920 (5.09%) | 113051423 (87.49%) |
| d0_2 | 89,340,406 | 88,801,486 | 91.8 | 82773403 (93.21%) | 4284549 (4.82%) | 78488854 (88.39%) |
| d0_3 | 99,160,794 | 98,336,878 | 90.59 | 91001761 (92.54%) | 4718019 (4.8%) | 86283742 (87.74%) |
| d4_1 | 121,235,508 | 119490,034 | 91.51 | 110289418 (92.3%) | 7582259 (6.35%) | 102707159 (85.95%) |
| d4_2 | 99,295,402 | 98,542,902 | 91.78 | 90935661 (92.28%) | 5696835 (5.78%) | 85238826 (86.5%) |
| d4_3 | 144,647,132 | 143,049,194 | 90.88 | 129004354 (90.18%) | 7046316 (4.93%) | 121958038 (85.26%) |
| d8_1 | 90,045,956 | 88,794,998 | 90.8 | 81566350 (91.86%) | 2499967 (2.82%) | 79066383 (89.04%) |
| d8_2 | 86,765,378 | 86,067,294 | 92.06 | 80143509 (93.12%) | 2977277 (3.46%) | 77166232 (89.66%) |
| d8_3 | 111,493,854 | 110,673,512 | 90.72 | 101662004 (91.86%) | 4964561 (4.49%) | 96697443 (87.37%) |
